# An Extended Review Concerning the Relevance of Deep Learning and Privacy Techniques for Data-Driven Soft Sensors

**DOI:** 10.3390/s23010294

**Published:** 2022-12-27

**Authors:** Razvan Bocu, Dorin Bocu, Maksim Iavich

**Affiliations:** 1 Department of Mathematics and Computer Science, Transilvania University of Brasov, 500036 Brașov, Romania; 2 Department of Research and Technology, Siemens Industry Software, 500203 Brașov, Romania; 3 Department of Computer Science, Caucasus University, Tbilisi 0102, Georgia

**Keywords:** data-driven, soft sensors, deep learning, mobile devices, background sensors, personal data, data privacy

## Abstract

The continuously increasing number of mobile devices actively being used in the world amounted to approximately 6.8 billion by 2022. Consequently, this implies a substantial increase in the amount of personal data collected, transported, processed, and stored. The authors of this paper designed and implemented an integrated personal health data management system, which considers data-driven software and hardware sensors, comprehensive data privacy techniques, and machine-learning-based algorithmic models. It was determined that there are very few relevant and complete surveys concerning this specific problem. Therefore, the current scientific research was considered, and this paper comprehensively analyzes the importance of deep learning techniques that are applied to the overall management of data collected by data-driven soft sensors. This survey considers aspects that are related to demographics, health and body parameters, and human activity and behaviour pattern detection. Additionally, the relatively complex problem of designing and implementing data privacy mechanisms, while ensuring efficient data access, is also discussed, and the relevant metrics are presented. The paper concludes by presenting the most important open research questions and challenges. The paper provides a comprehensive and thorough scientific literature survey, which is useful for any researcher or practitioner in the scope of data-driven soft sensors and privacy techniques, in relation to the relevant machine-learning-based models.

## 1. Introduction

Mobile devices and appliances such as various wearables, handheld smartphones, or tablets feature certain sensors that collect a large amount of personal information for a variety of scenarios and purposes [1,2]. Additionally, the significant increase of wearables’ computational capabilities makes them suitable for many real-world uses [3,4]. The ubiquitous availability of the mobile devices makes them obvious targets for cyberattacks designed to illegitimately gain access to personal data and resources [5].

This survey provides the following contributions to the literature.

- A comprehensive analysis of the personal data collected using mobile background sensors and the related machine-learning- and deep-learning-based automated methods that focus on sociological and demographic aspects.
- A presentation of the generally considered applications and real-world use case scenarios related to the use of mobile devices.
- An overview of the relevant sensors and the related raw data usually available in mobile computing environments and mobile devices. This particularly analyzes the background sensors, as they are usually perceived as harmless by the average end user.
- A presentation of the metrics introduced in the relevant scientific and technical literature.

The scientific approaches reported in [6,7] describe particular encryption scheme models [8] that relate to data privacy [9]. Thus, the acquired data are consolidated on the client side [10]. The algorithmic models for the sum aggregate and the minimum aggregate procedures are implemented using an additive homomorphic encryption scheme in [11]. It is important to mention that these basic operations accept a set of values as input and output one value. Furthermore, they designate flexible and computationally efficient algorithms [12,13], which are considered in various other contexts that imply data processing in an aggregated manner. Thus, let us consider the queries that are performed relative to relational databases, which are structured as tables with rows and columns. In this case, the data processing is designed to take place on the client devices considering the vast majority of the existing relevant approaches [14,15]. Nevertheless, the operations that relate to the homomorphic encryption schemes usually require significant computational resources [16,17]. The computational models that are reported in the existing literature are generally unsuitable for conducting arithmetic operations directly over the encrypted data in the case of large real-world use cases. Papers [18,19] examine the proper offloading of data processing routines to the cloud. Furthermore, ref. [20] described a data-privacy-preserving model that considers sum aggregation that conducts most of the data processing operations on the client devices. Moreover, ref. [21] described a homomorphic encryption model that conducts the sum operation. This is insufficient for most of the real-world use cases. Therefore, a mechanism that allows for basic arithmetic operations to take place directly over the encrypted data needs to be designed [22,23]. Such an approach is generally known as verifiable computation [24,25].

The mechanism of verifiable computation was described by Gennaro et al. [12]. It allows for data to be offloaded from mobile client devices through various third-party data processing applications. Moreover, the client devices are capable of verifying the accuracy of the data computation results that they receive [26,27]. The contribution that was presented in [28] relates to a verifiable computational model that considers the data input relative to a plain text format. Furthermore, ref. [29] proposed a data processing model that relates to homomorphic data aggregation processes in connection with eHealth information systems. It is important to note that this algorithmic approach is not capable of verifying whether the obtained results are correct, which represents a fundamental functional requirement of any privacy-preserving data processing approach. Furthermore, the article also described a publicly verifiable data processing model that relates to large polynomials and matrices. Consequently, ref. [30] described a verifiable delegated data processing scheme which is based on set structures and operations. Thus, this refers to a set union, a set intersection, and a set difference. It is important to note that these algorithmic schemes are compatible with input data that are generated in a plain text format [31,32].

The European Union enacted the General Data Protection Regulation (GDPR), which defines personal data as any information that pertains to an identified or identifiable natural person [33]. The GDPR also defines sensitive data as a subset of personal information that includes the following categories of personal data: personal data that reveal racial or ethnic origin, political opinions, and religious or philosophical beliefs; trade union membership data; genetic data and biometric data which is processed exclusively to identify a human being; personal health data, and also data that concern aspects related to sex life or sexual orientation [33]. The automated processing of personal user data, which is also designated as user profiling [33], may easily determine such attributes using data acquired from mobile devices. This may be determined by the request for irrelevant data access permissions, the unclear and weak definition of permission items, and the incorrect use of permissions. It is relevant to state that this is also determined by the improper aggregation of personal data [34,35]. The prevention of misuse is the objective of several Innovative Training Networks (ITN), such as PriMa [36] and TReSPAsS [37].

Thus, the discussion may consider the problem of general privacy protection and sensitive personal data protection [38]. The ultimate goal is to secure personal user data through a de-identification process and consequently prevent re-identification [39] of sensitive personal identifiers [40], such as names, addresses, social security unique identifiers [41,42], etc. Nevertheless, personal data privacy protection is naturally connected to the realm of cybersecurity [43]. In essence, the goal is to securely modify the data so that it cannot be read or understood [44,45], while allowing efficient data processing to take place in the case of legitimate requests [46]. It is also important to consider related discussion and research topics, such as the study of large databases, which can be assimilated in the category of big data stores [47].

This survey considers the significant perspectives of the General Data Protection Act (GDPR), article 21, which states that the subject shall have the right to object, on grounds relating to his or her particular situation, at any time to processing of personal data concerning him or her.

The rest of this paper is organized as follows. First, the methodological principles are fully described. Then, relevant details concerning proposals for full privacy-preserving models are discussed, while an overview of the sensors and the raw data usually considered in modern mobile devices is provided. Additionally, the most relevant real-world use-case scenarios are presented, and the problem of personal user data processing is examined. Furthermore, the methods that are useful to collect and process data are comprehensively analyzed, and the relevant metrics are described. Moreover, the general data privacy methods are discussed. The section that follows presents the authors’ analytical remarks relative to important research aspects and gaps which have been determined during this comprehensive research process. The last section concludes the paper and discusses certain problems that were encountered during our research.

## 2. Research Methodology

This survey paper provides a systematic review (SR) research model, which is structured using the methodology designated as “Preferred Reporting Items for Systematic Reviews and Meta-Analysis” (PRISMA) [48]. Thus, the literature review methodology considers the following stages: definition of research questions, research of proper scientific contributions, definition of the proper inclusion and exclusion criteria.

### 2.1. Research Questions

The survey considers the following reference research questions.

- What are the sensors and the raw data commonly available on modern mobile devices, paying special attention to background sensors, which are often considered harmless by the end users?
- What are the typical related real-world use case scenarios?
- What are the relevant practical purposes of the data that are collected using mobile sensors?
- What are the specific features and logical structure of the data, which pertain to the various analyzed real-world use-case scenarios?
- What are the most frequently used data privacy and anonymization techniques?
- What are the metrics that quantify the level of data anonymization processes?

The next subsection specifies the logical structure of the effective research process.

### 2.2. Research Process

This subsection specifies in Table 1 the sources that have been considered to determine and collect the proper scientific literature that was surveyed.

The following subsection describes the exclusion and inclusion criteria considered to further filter less relevant papers in an objective way.

### 2.3. Exclusion and Inclusion Criteria

The relevance of the reviewed papers, and consequently the scientific efficiency of this survey paper, is further ensured by several inclusion criteria (IC) and exclusion criteria (EC). Thus, contributions that do not meet the defined EC are discarded. The IC-based filtering process follows a logical process, which is structured according to the following steps.

- Step 1. Abstract-based filtering: irrelevant scientific contributions are ignored based on the information that is extracted from the abstract and also considering the keywords. Thus, papers that meet at least 50% of the relevance threshold are considered further.
- Step 2. Full text-based filtering: papers that address only a small part of the scientific scope, which is defined by the abstract and the keywords, are ignored.
- Step 3. Quality analysis-based filtering: the remaining papers are further filtered out if any of the following conditions are not satisfied: <The paper proposes a comprehensive solution regarding the usage of data-driven soft sensors.> AND <The paper thoroughly describes the technical implementation of the proposed solution.> AND <The paper reviews related similar scientific contributions.> AND <The paper discusses and analyzes the obtained results.>

Consequently, the reference inclusion criteria are described in Table 2.

Furthermore, the reference exclusion criteria are presented in Table 3.

The following sections thoroughly survey the vast scope of scientific papers, which were selected according to the principles of this scientific survey methodology.

## 3. Data Acquisition through Mobile Devices and Sensors

Mobile devices provide a comprehensive set of functional features, which may be used for the proper processing and collection of related data. As an example, modern smartphones are equipped with powerful hardware components, such as multicore processors, sophisticated mobile graphical processing units (GPU), several gigabytes of memory, and a comprehensive set of built-in sensors. Additionally, it is possible to add new sensors using the wireless and even wired connection features of these mobile devices. The following subsection presents relevant contributions, which pertain to the design and implementation of full privacy-preserving data channels. Moreover, the possibility to conduct arithmetic operations directly over the encrypted data is discussed.

### 3.1. Remarks Concerning Full Privacy-Preserving Data Computation

The authors of [16] reported a verifiable data processing model that is related to encrypted input data in connection with mHealth (mobile health) software systems. The algorithmic scheme that is designated as accumulation tree was reported in [17], which verifies the results of geographical proximity tests. Furthermore, ref. [18] described the results that relate to verifiable computation use cases, which pertain to encrypted input data. It is important to mention that most of the existing approaches consider data processing at the level of the client devices. This approach does not apply to integrated data management systems which consider personal private data.

The advantages of cloud-based data storage and processing are obvious [49]. However, the design of the proper data security approaches determines a significant problem that generates conceptual issues to the cloud service providers [20,50].

The implied service providers aim to design and deploy layered security mechanisms. Nevertheless, the plain text data may still be accessed and used through proper intrusion techniques. Consequently, data must be encrypted before transmission to the respective external data processing modules. The relevant reviewed papers suggest a significant computation overhead connected to the mobile client devices [21]. This is especially relevant for the personal mobile devices, which collect medical data processed by proper integrated software systems. There are, however, approaches [22] that do not specify proper data privacy mechanisms [23] when the data are transmitted through the respective data channels. The proper management of personal health information (PHI) data refers to ethical principles and formal regulations [24]. Thus, it is necessary to design and implement integrated data processing systems that consider all the relevant constraints. The authors of [25] described the general architectures and the life cycles of cloud-based data processing services.

Ever since C. Gentry first described the concept of homomorphic encryption in 2009 [6], significant research has focused on improving [26] this computationally expensive data processing scheme. Consequently, many relevant real-world use cases pertain to the use of proper powerful hardware resources [27]. Moreover, the initial homomorphic encryption approaches were particularly computationally expensive relative to the respective real-world use cases [28]. Furthermore, the algorithmic apparatus was improved through multiple development phases [29]. Some papers have reported improvements to the computational efficiency of homomorphic encryption. For example, refs. [30,31,32,33] expanded the initial set of algorithms. The algorithmic model presented in [34,35] and also in ref. [36] may be used during the design of data processing components that are part of relevant integrated data management systems [37]. It is important to note that the comprehensive validity evaluation that we conducted [34] proves that even certain improved homomorphic encryption approaches are not adequate [51,52] relative to the timely processing of the collected medical data on the client side [35,53]. Furthermore, it is important to note the papers [54,55] that are connected to the full scope of ubiquitous systems. Thus, ref. [56] described a software application defined by two functional requirements. First, the system is able to conduct the semantic analysis of data that are produced by user interactions, which are connected to various contextual parameters that determine usual activities of daily living (ADL). This has the goal of determining the relevant behavioral patterns that define complex activities. Moreover, the software system is based on an algorithmic routine that supports the decision-making processes. Furthermore, a relevant contribution is reported in papers [57,58]. Additionally, ref. [59] described a general architecture of a ubiquitous system that is compatible with general medical use case scenarios and data storage models, such as the ones that are described in papers [60,61]. Moreover, software systems defined by interesting architectural models are presented in papers [62,63], and also [64,65]. It is significant to note that the authors of papers [66,67], and also [68] propose technical solutions that are relevant for the implementation of distributed personal data processing systems, which use wireless data transfer channels. Furthermore, the survey effort that is included in [69] created interesting perspectives on related scientific problems. Moreover, the authors of [70,71] proposed interesting data transmission models relative to next-generation radio networks, while [72] described a versatile data communication channels management system, which can be used in a variety of real-world use case scenarios, including vehicular ad hoc networks (VANET).

Moreover, it is important to mention the contributions that were described in [73,74], considering that they presented one of the few existing integrated personal data management systems, which fully implements data protection mechanisms considering all the relevant stages: data collection, transportation, processing, and long-term storage.

The survey that was conducted suggests the following requirements for any suitable integrated personal data management system.

- The collection of personal data is conducted using mobile client devices.
- The data is transferred to central data processing components.
- The data are properly and securely stored, and privacy-preserving data is processed.
- The system should be specified considering a flexible and decoupled system architecture which would allow for an efficient extension and re-structuring of the system in the future.
- The legal and formal requirements that are formalized by American and European regulations are also considered.
- The efficient integration of the system in the target software frameworks considers the specifics of the respective use cases, as well as all the technical and legal requirements.

### 3.2. Analytical Remarks Concerning Similar Contributions

The theoretical and practical survey presented in this paper is complemented by an analytical evaluation of existing data privacy approaches. This is contained in the following paragraphs.

Thus, ref. [75] relates to a comprehensive review of similar data privacy mechanisms, with a focus on e-Health software systems. Relevant advantages and disadvantages of reviewed models are analyzed. The papers were selected considering the similarity that was observed in the reviewed literature. The authors also describe the general features of a technical standard, which may define an e-Health system. The paper also includes a taxonomy of cloud-based models, while the relevant personal data privacy and security requirements enforced by the Health Insurance Portability and Accountability Act (HIPAA) [76,77] are analyzed. It is important to note that the authors describe a secure and dependable system architecture, which is compatible with electronic health scenarios that could guarantee efficiency, reliability, and a properly regulated access framework to health information. The main drawback of this architecture is its inability to deploy on distributed and structurally scalable infrastructures. Additionally, only standard asymmetric encryption models are implemented, which do not provide the necessary degree of health data privacy.

The general scope of cloud-based healthcare computing has modified real-world healthcare in several ways. Cloud infrastructures provide a discernible advantage in the scalability of service, and the possibility to alter the related computational and data storage resources. Additionally, other articles examine the implied security and data privacy-preserving mechanisms. This is an important aspect of the overall research problem, as it determines important legal and technological aspects that should be evaluated. In this respect, ref. [78] examines several scientific approaches that miss at least some of the necessary technical features. Thus, it is important to mention the end-to-end private data transmission channels, the mandatory scalability, and the architectural compatibility of diverse technical platforms and frameworks, which concern the implied client and back-end (server) components.

The obvious advances in the field of information and communication technology naturally relate to an improved economic environment that offers higher-value services to consumers and businesses. The health sector benefits from this progress. Although the cloud-based system architectures provide clear advantages, the remaining security and data privacy issues should still be considered and addressed. Thus, ref. [79] presented a distributed system that considers various data security levels and data encryption models. This heterogeneous architectural structure implies administrative, functional, and data security problems that suggest that the reported approach is not suited for real-time deployments of large-scale medical data processing systems.

The continuous development of Internet of Things (IoT) as a theoretically and practically relevant paradigm, which has occurred during the past twenty years, implies that novel personal data management approaches may be developed. Thus, ref. [80] presented an important problem, which concerns the fully secure preservation of personal data privacy. The article proposes an access control mechanism for cloud-based data that follows a certificate-based authentication model. The authors describe the methodology of the approach using the results of experimental evaluation processes. This suggests an apparent enhancement of the overall system’s security and performance through the optimization of the time needed to specify and implement the data and service access permissions. Nevertheless, the proposed approach does not offer the necessary scalability or end-to-end private data transmission channels between the client devices and the back end data processing components.

Significant progress has been made in the scope of cloud-based healthcare applications in the past ten years, particularly due to the implied remote access features, among other advantages. It is important to note that the reviewed literature demonstrates the resistance of certain end users to the adoption of the new technologies, particularly in developing nations [81]. This article suggests that user experience constitutes another significant perspective, which should be considered in any research. Moreover, personal data collection should occur in a seamless manner without any costly modifications to client mobile devices. In this context, the contribution that is reported in [82] proposed a rather interesting data analytics framework, which considers regressive machine learning techniques and Internet of Things (IoT) devices in relation to the field of precision agriculture.

Attribute-based encryption (ABE) models represent an interesting use case in healthcare. Patients encrypt their electronic health record (EHR), assign the attributes, and send them to the cloud. Healthcare professionals receive the encrypted EHR corresponding to their field of expertise from the cloud-based system. Decryption of the EHR data presumes that the medical personnel receive the secret keys from the key generation center (KGC). Thus, the KGC stores the secret keys of all the encrypted EHR records. Consequently, it is possible to decrypt the relevant patients’ records, which represents a security issue. A decentralized ABE scheme addresses this issue, but it implies significant computation and communication costs. Furthermore, unauthorized medical employees may be able to read the patients’ private EHR data. Additionally, the privacy of the KGC’s secret keys and the doctor’s attribute privacy determine relevant research aspects. Thus, ref. [83] presented a cloud-based privacy-preserving e-health (CP2EH) scheme, which addresses the issues of unauthorized access to patient records and the proper management of the doctor’s attribute privacy relative to an ABE scheme. The presented model includes the oblivious transfer (OT) and zero-knowledge proof (ZKP) protocols in the centralized ABE scheme. Thus, the OT protocol ensures the privacy of the secret keys and the doctor’s attribute. Despite the reported advantages, the system is selective concerning the accepted data acquisition devices. Moreover, it is compatible with only certain software frameworks, it does not scale well, and it does not implement end-to-end secure data transmission channels.

The authors in [84] presented an attribute-based encryption (ABE) access control model. This enforces controlled and possibly multi-level access delegation policies. Moreover, the authors evaluate the possibility of deploying such a system in an e-health environment with the goal of safely sharing EHR data of the patients enrolled in the system. The authors assert that the proposed mechanism is safe from some plaintext attacks and from attacks based on attribute collusion [1]. Although this appears to be one of the most promising approaches that we reviewed, it also does not provide end-to-end private medical data transmission channels. Moreover, it manifests the fundamental architectural and functional problems, which have already been enumerated.

The general problem that is approached in this paper also pertains to particularly relevant real-world use cases, such as smart grids, which are studied in certain interesting papers. Thus, ref. [85] intends to present an analysis of research trends that pertain to smart grids. It describes a next-generation smart grid, which is based on the utilization of artificial intelligence (AI), Internet of Things (IoT) devices, and 5G data networks. The paper also comments on the conceptual and practical challenges that this modern architecture faces.

Our thorough literature review proves that although interesting contributions are described in the literature, most of the existing algorithmic and functional models miss some of the mandatory technical features. In contrast, he integrated medical data management system described in [74] represents one of the few approaches that fulfills all of the necessary algorithmic and technical constraints.

## 4. General Mobile Collection of Sensitive Data

Mobile devices possess the hardware capabilities needed to facilitate general data collection and processing. These include powerful multicore central processing units (CPU), graphical processing units (GPU), and random access memory (RAM) which sustain powerful and versatile operating systems. The mentioned hardware and software features support efficient data sensing and collection operations, together with the usual smartphone core functions.

Consequently, the built-in mobile sensors are able to collect data considering an adequate frequency for the data acquisition interval and for the private data categories.

The remarks are applicable to many types of mobile wearable devices, such as smartwatches, which can be assimilated to the wider scope of Internet of Things (IoT) devices, as long as they are connected to the Internet or are linked to devices that are directly connected to the Internet [86]. These devices are rapidly becoming capable of performing complex measurements, and even local data analysis processes [87]. In principle, mobile device manufacturers implement and provide the required mobile applications, which can be installed on their wearable devices. Nevertheless, although these mobile applications are adequate for general use case scenarios, specialized applications are required to sustain specific real-world scenarios.

**Figure 1:**
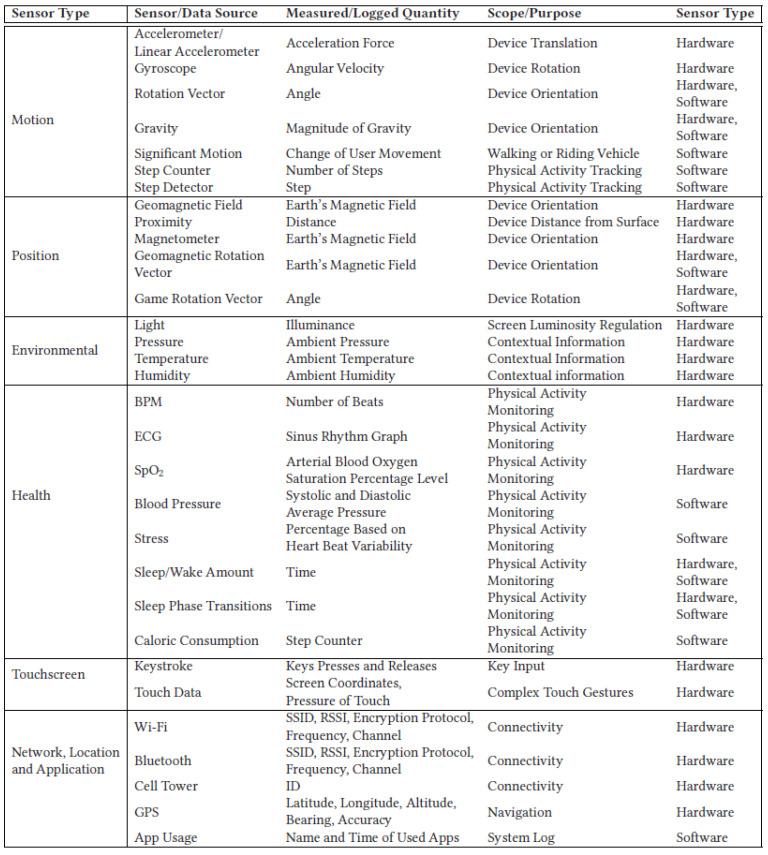
Sensors and raw data specific to modern mobile devices.

Figure 1 presents the sensors and raw data types used in mobile devices. The sensors can be grouped in two categories depending on whether the output signal is generated using hardware or software. The hardware sensors translate physical measurements into electrical signals that are converted to a digital format to be processed. The software sensors use the data previously generated by the hardware sensors to perform the necessary computations.

Motion sensors are designed to measure both the rotational and acceleration forces over the three axes of the related device. Thus, the hardware motion sensors keep track of the angular velocity and acceleration, and the software sensors may produce an output according to either a continuous or an event-driven pattern. Moreover, the position sensors imply the measurement of changes in the Earth’s magnetic field related to the actual physical orientation, while environmental sensors are typically activated by an event and return a value measurement in the form of one scalar. These sensors may be configured to return continuous measurements, which may attain a frequency of approximately 200 Hz, while their power consumption is still kept at a low level [75].

Certain measurements that discern biological and physiological parameters are supported on particular mobile devices due to specialized health sensors. As an example, many mobile devices, including smartwatches, feature optical sensors used to detect the changes in the volume of the blood flowing through the arteries. Consequently, physiological heart parameters are evaluated. Additionally, studies that pertain to other health problems, such as sleep disorders in the scope of polysomnography, also use sensors [88,89].

Data generated by user interaction with a touchscreen can be quantified by the number of “keystrokes” [90] or by analyzing the touch data generated by the user [91]. Considering the former situation, the virtual keystrokes are recorded, and the timestamp and pressure data are also logged for each keystroke. The acquired data allow analysts to discern even more complex features, such as the time between keystrokes, the time allocated for touch and hold operations, and so on [92]. Supplementary to the actual keystrokes, modern touchscreen panels significantly expanded the user interaction area, which includes the screen zones that are sensitive to user touch operations. Thus, it is possible to precisely determine the location of the touch points using a coordinate system relative to the X and Y coordinates of the screen. Additionally, all of the other usual gestures, such as pinch, tap, swipe, multitouch, and more advanced user interaction parameters, such as angle, velocity, trajectory, and acceleration, can be extracted [93].

Data connections represent a basic but fundamental aspect of mobile devices that imply the implementation and full or partial compatibility with a vast set of network protocols. The networked data connections generate private data patterns concerning the user’s daily patterns. Consequently, they can support the profiling of human behaviour and the acquisition of related sensitive personal data. Considering that the 5G radio standard is currently during its early stages of commercial deployment and that the 6G radio standard is under development, it can be asserted that the improved data transfer rates and the significantly lower latency values will expand the functional capabilities of machine-to-machine (M2M) communications. This should essentially increase the research and commercial relevance of mobile devices [94].

## 5. Real-World Sensors Use Case Scenarios

The two mainstream mobile operating systems, Android and iOS, initially offered less than 500 applications for download in their application stores. Currently, Google Play, which represents the Android applications store, includes over 3.5 million applications, while Apple’s App Store offers approximately 2.2 million applications. It is also interesting to note that Amazon App store contains approximately 500,000 applications [95]. The extensive range of applications cover various use case scenarios, the most relevant of which are discussed in the following paragraphs.

### 5.1. User Authentication Systems

Considering the mainstream user authentication systems, legitimate users are required to provide a secret token, such as a password or a personal identification number (PIN) code. This authentication model is commonly known as “what you know”. Moreover, there are authentication systems based on certain physical items, such as public key infrastructure (PKI) cards, which are known as “what you have”. Additionally, other authentication systems consider users’ physical features, such as fingerprints or geometry of the eyes, to perform the authentication. This is known as the “what you are” paradigm [96].

Biometrics are common and fundamental instruments of mobile authentication systems. The biometrics may belong to both physiological and behavioral categories [97]. As an example, we may speak about entry point fingerprints, or face-based identification. Nevertheless, using such authentication models implies that the device remains unlocked and accessible, and any unauthorized access is possible. This shortcoming may be averted through continuous authentication schemes relative to mobile devices, which use behavioral biometric authentication mechanisms [98]. Thus, the biometric data are continuously collected through a passive model during normal use of the mobile device, which ensures that the user’s physical features correspond to those of the legitimate owner. Nevertheless, several logical or environmental features, such as scenarios, modalities, or environmental traits, may adversely influence the accuracy of mobile biometric systems [99]. Thus, the literature reports hybrid solutions, which combine background sensors [100,101], touchscreen devices [102], and network information [103]. This supports the development of higher-accuracy continuous authentication systems, which are based on behavioural biometric mechanisms.

### 5.2. Fitness and Healthcare Systems and Services

Mobile applications and devices play an important role in the healthcare sector. Thus, “mHealth” (mobile health) is a concept referring to a subset of eHealth that encompasses medical and public health procedures supported by mobile devices. Mobile applications support the general healthcare processes. Thus, patients may avail themselves of improved and more efficient services regarding acute and chronic conditions [104].

Mobile applications can represent real-world use cases, which are capable of analyzing body postures and generating reports concerning mental disorders [105]. They may also monitor medical conditions, such as Parkinson disease, stress, dementia, among others [106,107]. Furthermore, mobile health applications may support the improvement of a healthy lifestyle. Thus, a variety of mobile devices, such as mobile phones and smartwatches, are used to track the intensity of the measured physical activity, including all the relevant physiological parameters [108,109,110].

Additionally, existing scientific studies report integrated mHealth and eHealth software systems that support the collection of personal health data using mobile and wearable devices, the processing of the data components, the format of the encrypted data to conduct arithmetic operations, and secure-long term personal health data storage. This type of full privacy preserving approach, which relates to homomorphic encryption and virtualized 5G data channels, was described in [73,74].

### 5.3. Services Based on Location Data

Mobile devices fetch geolocation data using several sources, including the Global Positioning System (GPS) hardware devices. These data are used by mobile applications to determine the geographical position of the users for a variety of purposes, such as navigation hints data or targeted advertising [111]. The applications that consider geolocation data (location-aware applications) belong to the realm of the context awareness paradigm [112]. Moreover, radio protocols used to transmit data short distances, such as Wi-Fi (Wireless Fidelity) and Bluetooth, allow the mobile devices to exchange data with neighbouring devices and consequently use them for their purposes. This approach may be used to specify a semantic context, which is determined by the immediate environment. As an example, the contribution that is reported in [113] described the specification and implementation of virtual tours in museums, which would provide relevant information to the visitors based on the neighbourhood of the visitors’ actual position in the museum. Furthermore, interesting relevant aspects may also be studied in [114].

### 5.4. Remarks Concerning Other Relevant Use Cases

Considering the mainstream use cases, background sensors improve the end users’ experience in various ways. As an example, the determination of a mobile device’s position is facilitated by the background sensors, which implement the automatic change of the screen orientation. Obviously, data generated by the light sensors support the automatic adjustment of the screen brightness. Moreover, the proximity sensor manages the screen lock or unlock states in different situations, for example when placing a phone call. Another interesting use case is represented by the augmented reality (AR) applications in fields such as entertainment, commerce, and navigation [115]. The AR applications rely essentially on the data generated by the background sensors.

The ubiquity of modern mobile devices allows for more complex but useful real-world use case scenarios, such as mobile participatory sensing [116]. Thus, particular users voluntarily agree to share their devices to collect data that are relevant for the analysis of various aspects of the implied reality. This mediates the collection of relevant data, which are consequently used to assess, measure, and map various phenomena through a crowd-sourced participatory manner [111]. These use case scenarios include, among others, monitoring urban noise and pollution levels, monitoring urban cleanliness levels, and monitoring urban road and traffic conditions [117].

## 6. Proper Management of Sensitive Private Data

The automated management of data collected through mobile device use involves interaction with an appreciable amount of sensitive private data. It is important to note that some mobile sensors, such as GPS hardware components, microphones, and cameras, are especially difficult to tamper with, as they require special access permissions. Nevertheless, other mobile sensors, devices, or resources, such as the touchscreen, accelerometer, and networking data logs, require a lower level of access permission. Additionally, these data may be used to create a backdoor to sensitive personal data, considering that they can be sufficient to re-identify a particular individual through attributes, such as personal health data, particulars of daily routines, or demographic data.

The intimate nature of sensitive personal data requires the design and implementation of particular secure data management mechanisms. The most defining trait of this type of data relates to its uniqueness relative to the respective individuals. This is particularly relevant in relation to biometric data. Considering the wider scope of biometrics research, the main research and development challenges are represented by the mechanisms for storing personal data, the administrator or owner of the implied software and hardware data processing system, and the biometric features used to perform the authentication. Furthermore, the type and time reliability of the considered biometric features also represent a relevant question [118]. The next subsections discuss on the most relevant types of sensitive personal data that can be generated by the mobile devices’ sensors.

### 6.1. Demographic Data

Arguably, the most prevalent type of sensitive personal data is demographics, which includes attributes such as ethnicity, age, or gender.

#### 6.1.1. Sensors That Detect Movement

The authors in [119] considered the determination of a user’s age range using data generated by an accelerometer. This was achieved during an experiment that involved performing a preset series of taps on a touchscreen relative to several contact spots. The experiment used the k-nearest neighbor (k-NN) algorithm, which produces an accuracy of 85.3%. Moreover, the authors of [120] reported an algorithmic mode that discriminates an adult from a child through behavioural particularities captured by the mobile motion sensors. The main hypothesis states that children, who have smaller hands, are shakier. The algorithmic model produced an accuracy of 96% through the random forest (RF) approach. The scientific contribution reported in [121], obtained the gender of the end users by analyzing their their walking routines data, which were collected by mobile motion sensors. The proposed model produced an accuracy of 76.8% using support vector machines (SVMs), and bagging algorithms. Moreover, the authors of [122] described an approach for recognition of gender data using gait (walking) data, which were collected by the mobile sensors. The reported accuracy was 96.3%, and the process used the bagged tree classifier.

The authors of [123] reported an automatic gender recognition algorithm, which uses the data collected by a gyroscope and accelerometer. The generated accuracy was 80% using the principal component analysis (PCA) technique. Moreover, the authors of [124] determined gender and age data using hidden Markov models (HMMs). Thus, the authors set up a competition which compared data collected by an accelerometer with gyroscope data using the respective mobile devices. The reported error percentage was 24.23% relative to the gender and 5.39% relative to age. The notable progress in the field of deep learning enhanced the results, as was the case with the findings described in [125]. Thus, the authors mentioned an accuracy of 94.11%, which was obtained through the analysis of gait (walking) data as it related to gender classification. The authors used long short-term memory (LSTM) and recurrent neural networks (RNNs) which are suitable for capturing the temporal dependencies that defined by the analyzed data.

#### 6.1.2. Touchscreen Data

In [126], the authors categorized end users in two categories, adults and children, based on the mechanics of tap and swipe gestures. The authors describes an active user detection (AUD) algorithm, which generates an accuracy of 97%. Furthermore, ref. [2] presented a database that stores childrens’ mobile interaction data. The considered touch interaction data allowed the children to be assigned to three categories, which included ages from 18 months to 8 years. The described model was based on the support vector machine (SVM) technique and yielded an accuracy of 90.45%. Furthermore, the authors of [120] reported a study based on the random forest (RF) technique, which used the tap gesture data to distinguish between adults and children. The model functions with an accuracy of 99%. Other papers report on using touchscreen data to determine an individual’s gender. The study reported in [127] considered the prediction of soft biometrics data generated by swipe gestures. The measured accuracy was 78%, which was based on a decision voting scheme determined by four distinct classifiers: decision tree (DT), naive Bayes (NB), support vector machine (SVM), and logistic regression (LR). The authors of [128] collected behavioral data using mobile devices’ accelerometers, gyroscopes, and orientation sensors, which were activated during the end users’ interactions with their mobile devices. The gesture data, which determine the gender of the user, were processed using a k-NN classifier with an accuracy of 93.65%.

#### 6.1.3. Sensor Data Related to Mobile Applications, Location, and Network

Research has proven a correlation between geolocation data and the end users’ demographics and usage patterns. As an example, in [129], the researchers stressed the significance of data generated by mobile devices in the context of demographic modeling and data measurement, while circumventing the need for traditional censuses and sociological research. This approach significantly speeds up the related political decisions. Furthermore, the authors of [130] considered radius, eccentricity, and entropy as three parameters that define travel behavior. More precisely, the authors attempted to explain the correlation between mobile device use and personal travel behaviour, which further analyzes the correlation between the frequency of the phone calls, and certain demographic factors, such as age, gender, and the defining features of the environment.

Moreover, ref. [131] described an unsupervised, data-driven model designed to create user categories that consider high-resolution mobility data, which are acquired through mobile navigation applications. The contribution reported in [132] described a method for the inference of demographic information using social networks photos, which include geographic tagging data. More precisely, this shows how an individual’s ethnic characteristics can be obtained from collected geolocation data related to two particular metropolitan zones. The described model determines three ethnic groups, and the accuracy was reported as 72% using logistic regression (LR).

The scientific contribution reported in [133] discussed the suitability of geolocation data in inferring information regarding marital status and actual residence. The described research process considered the determination of spatial and temporal features using human mobility patterns, together with other features related to the geographical context. This approach offers information concerning the places visited by the individuals under analysis, such as private homes, hospitals, or leisure facilities. The obtained accuracy was 80% based on an eXtreme gradient boosting (XGBoost) algorithm [134]. The scientific presentation in [135] started with an analysis of gender-related behavioral patterns determined by mobile applications, which are related to the use of Wi-Fi and Bluetooth. The authors reported on the possibility to predict the gender of the end users and showed an accuracy of 91.8%. The algorithm used random forest (RF) and multinomial naive Bayes (NB). The data were collected from network connection logs, and the events were sorted according to occurrence frequency. An assessment of the temporal patterns was conducted relative to the 1000 events that occurred with the highest frequency. This type of contextual behavioral information is particularly useful in various domains, such as advertisement customization and the personalization of home screens.

### 6.2. Remarks Concerning the Study of Human Behaviour

The literature proves that the general patterns of users’ daily activities and behavioural traits can be inferred from the data collected by mobile sensors [136]. This generates obvious problems regarding the privacy of the collected personal data, which should be properly addressed by academic and industrial research projects.

#### 6.2.1. Motion Sensors

The authors of [137] described a system that is able to assess an individual’s spatial mobility status. Thus, it can evaluate whether the person is stationary, walking, running, riding a bicycle, climbing stairs, going downstairs, or driving using only the accelerometer information. Their algorithmic approach, which is based on a support vector machine (SVM) technique, functions with an accuracy of up to 93.2%. Furthermore, the authors of [108] used mobile gyroscope and accelerometer data and developed an application used to track the user’s daily routines. Their model is based on a decision tree (DT) classifier, and the average area under the receiver operating characteristic (AUROC) curve was over 99.0%.

The authors in [138] considered users’ mobility while they were eating, as they are detected by the accelerometer sensor installed on smartwatches. The authors of [139] performed a classification of human drinking behavior. This took into account the data acquired by the accelerometer sensors of the mobile phones young adults used during nightlife activities. The accuracy of 76.1% was based on a density-based spatial clustering of applications (DBSCAN) algorithm. The respective approach also assessed the amount of ingested alcohol.

The assessment of user mood and physical state (sober, tipsy, or drunk) was conducted using the approach reported in [140] using accelerometer data. It also included a channel for users to report their own behaviour. Naturally, this was an auxiliary feature, which may not be regarded as an objective source of data. The algorithmic core was based on the random forest (RF) model, with an accuracy of 70%. Furthermore, mobile motion sensors were also used to collect data related to sleep, such as sleep habits and postures. The contribution that was reported in [141] uses accelerometer, gyroscope, and orientation data, which are retrieved using a smartwatch to detect and assess sleep postures (supine, left lateral, right lateral, prone). The reported algorithmic model produced an accuracy beyond 95%, which considered Euclidean distances. The described approach also evaluated the position of the users’ hand considering the following three states: placed on the abdomen, chest, or head. The described model used a k-NN algorithm, with an accuracy greater than 88%.

#### 6.2.2. Sensor Data Related to Mobile Applications, Location, and Network

The authors of [142] used GPS data to assess whether the user was standing, walking, or using other means of transportation. The algorithm used a fuzzy classifier, which calculated the speed and angle of the person relative to the ground. The measured accuracy was 96% considering the data, which were collected at five-second intervals. Additionally, it is also important to note that radio receivers and transmitters, by their nature, are also capable of providing information about users’ behavioural patterns. This is also susceptible to generating sensible personal data security issues, which should be addressed. Thus, ref. [143] used the received signal strength indicator (RSSI) to determine user activity types. These were selected from the following set of states: lying down, falling, walking, running, sitting down, and standing up. The algorithmic model used a convolutional neural network (CNN), and the accuracy rate was 97.7%. The authors of [144] used three neural networks relative to the channel state information (CSI), which was measured by the Wi-Fi module. This technique can allegedly determine whether an individual is sitting, standing, or walking with an accuracy rate of 83%.

### 6.3. Remarks Regarding Body Features and Health Parameters

#### 6.3.1. Motion Sensors

The body mass index (BMI) is a mathematical ratio that correlates the body mass and height of any person. The classic modality to compute this index is providing weight and height using the formula to calculate BMI. Human gait or style of walking is sustained by the synergistic cooperation established between hundreds of muscles and joints. Consequently, mobile motion sensors are capable of discerning various muscle movements, which are transformed into specific patterns for the traits of the individuals, such as BMI. Thus, the authors of [145] proposed a hybrid model based on a convolutional neural network and long short-term memory (CNN-LSTM) architecture. This is able to estimate BMI using the data generated by the accelerometer and the gyroscope, and the maximum determined accuracy is 94.8%. Considering BMI as a reference, several other health attributes may be determined [146,147]. It is interesting to note that another physiological variable that can be evaluated using accelerometer data is the level of stress. Thus, the authors of [148] reported an accuracy of 71% using the mentioned techniques and also the naive Bayes algorithm.

#### 6.3.2. Remarks Concerning the Touchscreen

Data generated by mobile sensors may be used to assess, even to diagnose, certain medical conditions. Thus, it is possible to determine whether a person suffers from Parkinson’s disease through the analysis of the respective users’ keystroke writing pattern, which is totally independent from the actual content of the text. The authors in [149] considered an SVM algorithm, which determines an area under the receiver operating characteristic (AUROC) of 0.88 relative to this particular problem. Furthermore, ref. [150] assessed several types of features, which are specified relative to various handwriting patterns. These are used as biometrics to study Parkinson’s disease. Moreover, in [151], the authors demonstrated that people with longer thumbs require less time to conduct swipe gestures.

#### 6.3.3. Sensors Data Related to Mobile Applications, Location, and Network

The authors of [152] described an application that detects periods of psychological depression using geolocation patterns, which are retrieved from the mobile devices of individuals with bipolar disorder (BD). The model uses a linear regression algorithm, together with a quadratic discriminant analysis algorithm. The method produced an accuracy of 85%. GPS data may also be used to detect various sleep disorders, such as sleep–wake stages and sleep-disordered breathing disorders (SRBD), such as obstructive sleep apnea (OSA). The model uses SVM algorithms and demonstrated accuracy of up to 92.3% [153,154]. StayActive3 is an application that detects stress by analyzing the behavior of users via smartphone, using the data from the Wi-Fi, step counter, location, and battery level, among others. It is also worth mentioning the software system, which is referred to as StayActive [155]. The authors used a combination of simple relaxation scores that relate to the information acquired from the sleeping patterns of enrolled users. This analysis measures the longest time intervals during which the enrolled end users did not touch the screen, the patterns of their social interaction, and physical activity to evaluate the level of the stress.

### 6.4. The Detection of Psychological Mood and Emotions

End users’ daily activities are dependent on their psychological mood. Consequently, valuable related data may be collected by various sensors.

#### 6.4.1. Motion Sensors

The authors of [156] researched the influence that mood may have on the recognition accuracy rate of mobile biometric systems. Thus, by using an RF classifier, the authors discovered users with face recognition accuracy less than 70% exhibited the fewest psychological mood changes. The accelerometer provides useful data concerning users’ walk patterns, which can be used to assess psychological mood relative to the following three states: happy, sad, or neutral. It is worth noting that the authors of [157] assessed mood using an RF algorithm, which produced a mean AUROC of 81%.

#### 6.4.2. Touchscreen Data

Many studies demonstrate a correlation between users’ interaction patterns with the screens of their mobile devices and their psychological mood. Thus, the authors of [158] researched the development of psychiatric diseases using an unobtrusive setup deployed in the patients’ personal environment. The process explored the connection between bipolar affective disorder syndrome and the use of mobile devices. Considering the data generated by keystroke metadata and the accelerometer sensor, they obtained a detection accuracy of 90.31% relative to the proper detection of psychiatric conditions. The findings reported in [159] described a preventive medical treatment recommendation system, which may be useful to prevent the actual onset of clinical depression. Thus, the authors presented a mobile application, which was used to acquire the users’ psychological states through the analysis of data provided by the call logs and the applications’ usage history. The model produced an accuracy score of 86%.

The analysis of finger strokes patterns during games [160] can help distinguish between four emotional states: excited, relaxed, frustrated, and bored. The SVM algorithm produced an accuracy score of 69%. Moreover, the findings reported in [161] analyzed the pattern of finger strokes as an indication of the end user’s psychological state, which can be classified as on e of three possible values: positive, negative, or neutral. The detection performed with an accuracy of 90.47% relative to a linear regression model.

#### 6.4.3. Sensors Data Related to Mobile Applications, Location, and Network

MoodExplorer is an application that collects data using various mobile sensors, such as GPS, accelerometer, and Wi-Fi components [162]. The authors inferred the correlation established between psychological states, which were reported by the end users themselves, and the usage patterns of the respective mobile devices. The reported approach determines five types of emotions: happiness, sadness, anger, surprise, and fear. The algorithmic model is called Graph Factor. The performance was evaluated using a metric designated as “match”, which featured an average value of 62.9%.

### 6.5. User Tracking through Location Data

Although mobile devices often feature dedicated GPS location devices, it is possible to determine geographic location using the data generated by other mobile sensors.

#### 6.5.1. Motion Sensors

Certain scientific articles demonstrate that the geographic location of a person can be determined using data generated by several mobile sensors, such as accelerometer, gyroscope, and magnetometer, during the person’s daily routines that involve using public transport, walking, or driving. The authors of [163] comparatively analyzed pre-defined routes, which were used by the end users relative to different means of transportation, such as walking, train, bus, or taxi. They compared the routes using a dynamic time warping (DTW) algorithm, which generated a Kullback–Leibler distance of 0.00057 relative to a taxi trip.

The authors in [164] described a modality that uses accelerometer data to track the end users’ underground routes. The generated accuracy was 92% considering six visited underground stations, which were based on boosted naive Bayes (NB), and decision tree (DT) algorithms. The authors of [165] proposed an algorithmic model that determines the geographic location of vehicle drivers using the data generated by mobile motion sensors. The described approach considers an approximation of the related trajectory using accelerometer data. The map coordinates are correlated with the approximated trajectory to generate precise geographic location data. The approach that is presented allows for the end user to be located with a maximum error of 200 m. The distance is calculated as the radius between the center of the circle, which represents the actual person’s location, and the approximated geographical location.

#### 6.5.2. Sensor Data Related to Mobile Applications, Location, and Network

The end users’ geographic location may also be determined using the data that identify encountered Wi-Fi networks. Thus, the authors of [166] described the indoor determination of the end users’ location in a real-time fashion. The geographical location determination was conducted with an accuracy of 85.7% through the utilization of a random forest (RF) algorithm.

### 6.6. Logging Keystroke Data and Text Inference Using Motion Sensors

Touchlogger [167] is an application that aims to detect the precise zone of the screen that is touched. The process considers the device’s micromovements as they are detected by the mobile gyroscope and accelerometer. The proposed approach considers a division of the screen into ten zones, which are analyzed using a probability density function relative to a Gaussian distribution. The application has shown an accuracy of 70%. It is also possible to determine the text that the end user generates based on the screen zones that are touched.

Furthermore, ref. [168] a related system has an accuracy rate of 93%, by utilizing a hierarchical classification scheme. Additionally, ref. [169] describes a controlled environment, which is used to detect various text patterns that are entered using the mobile devices’ touchscreen. Thus, the PIN code was correctly identified in 43% of the cases, while the unlock pattern was correctly detected in 73% of the cases. The algorithmic core is based on a hybrid model, which considers logistic regression (LR), and hidden markov models (HMM).

## 7. Metrics Related to the Privacy of Personal Sensitive Data

The general class of privacy and data de-identification methods essentially modify or remove original personal sensitive data. As an example, the practical usefulness of this data should be maintained by preserving the personal attributes, which do not offer any indication about the identity of the related individuals. As an example, the gender may be preserved, while names, surnames, or social security numbers should be removed or de-identified through various anonymization techniques [170]. There are numerous domains that require the design and implementation of proper anonymization techniques [47]. The suitability and practical efficiency of these data anonymization models are quantified using certain metrics, which are presented in the following subsections.

### 7.1. General Considerations

Sensitive personal data protection models are assessed by measuring the degree of data protection obtained and the remaining data utility after anonymization or de-identification procedures are applied. The former measurement is conducted using specific data privacy metrics, while the latter task is accomplished considering the quantitative decrease of classic metrics, such as accuracy or equal error rate (EER) [171].

Sensitive personal data collected using mobile sensors can be grouped in two fundamental categories. One is represented by structured data, such as high-level health data, networking data, geographical location data, and data generated by mobile applications. The other is unstructured data, which includes physical position data, environmental data, or low-level health data. Therefore, several metrics are necessary to properly evaluate the de-identification process relative to the particular real-world use case or problem domain. The following subsections present relevant aspects concerning proper data privacy assurance processes.

This paper examines the classification of privacy metrics considering the features of the determined output. More precisely, it is important to observe the features of processed data in relation to the respective metrics. It is also important to note that there is no universal metric that can be applied to all features. Therefore, several reported scientific studies define and use their own metrics. The following paragraphs examine the properties measured as the main classification criterion in some studies. According to this criterion, some of the most important privacy metrics in mobile devices can be grouped in the categories described in the following paragraphs [172].

#### 7.1.1. Metrics That Relate to Data Anonymity

Some of the related metrics originate into the basic model of k-anonymity [173]. This is defined as the impossibility to identify a certain individual from another k − 1 individual, provided that the relevant information is released. Sensitive private data are grouped into equivalence classes that include at least k individuals, which cannot be distinguished from their sensitive personal attributes. It is important to note that k-anonymity is independent of the particular technique used for the extraction of personal data. This is also useful to quantify the degree of similarity between the original and the de-identified datasets. Nevertheless, k-anonymity has some limitations, which have led to the development of new metrics based on the original anonymization model. The new metrics are aimed at overcoming some of k-anonymity’s issues by imposing additional requirements.

Thus, m-invariance [174] customizes k-anonymity to allow the processing of multiple versions of the same dataset. Moreover, (α, k)-Anonymity [175] specifies a threshold for the maximum occurrence frequency relative to the sensitive attributes that are part of a class to prevent the disclosure of essential attribute data. Furthermore, L-diversity [176] is designed to block linkage attacks by specifying the minimum diversity inside an equivalence class.

For a malformed set of sensitive attributes, t-closeness [177] and stochastic t-closeness [178] are described They consider the principle that the distribution of private values that belong to an equivalence class must be as similar as possible to the values’ distribution at the scale of the entire dataset. Furthermore, the characteristics of the original distribution are required to compute this metric. Moreover, considering the initial distribution of the data, the (c, t)-isolation [179] refers to the number of data samples, which exist near a data sample that is inferred from the de-identified data. It is relevant to mention another data anonymization model, (k, e)-anonymity [180], which concerns the semantic of the distance that exists between private user data items. This requires the data range of private data attributes that belong to any equivalence class to be greater than a precalibrated “safe” value.

It is important to note that in spite of the mentioned shortcomings, the family of k-anonymity metrics is actively used today in various real-world use case scenarios, especially for low-dimensional structured data [181]. This is justified by the inability of k-anonymity de-identification methods to provide a sufficient degree of data privacy in the case of high-dimensional data.

#### 7.1.2. Differential Metrics

Differential privacy is a fundamental technique used in the scope of data anonymization processes. It states that the individual user will not be affected in any adverse manner as a consequence of their sensitive personal data usage in any research study or other type of analysis, regardless of the existing experimental or scientific data available [182]. Thus, the implementation of differential privacy mechanisms is normally performed through the addition of noise to the original clear text data. Consequently, it is necessary to have access to the original data, which are not de-identified. Initially, differential privacy was proposed in the realm of databases as a technique that would prevent the various database queries outcomes to be distinguished. Consequently, it has been adapted to other real-world use cases that involve the usage of low-dimensional data, such as biometrics and machine learning systems. Thus, irrespective of the presence of a particular data subject, the probability for any sequence of query responses to occur is determined by a parameter that we can define as ϵ. This is calibrated considering a proper balance between the degree of data privacy and the possibility to use the de-identified data. Relative to a specific computational context and a certain value of ϵ, various differential algorithms may be used, which generate variable levels of accuracy.

In a similar way to the referenced k-anonymity method, the differential privacy model determines several relevant metrics, which include approximate differential privacy that has weaker privacy guarantees but nevertheless ensures a greater usefulness of the de-identified data [183]. Joint differential privacy [184] determines the software systems, which are characterized by private data subjects that can access their own data items but not other persons’ data items. Thus, relative to the privacy of geographical location data, geo-indistinguishability [185] is determined by the addition of noise that complies with the differential privacy requirements for a particular geographical location within a specified distance. Furthermore, computational differential privacy [186] involves a weaker adversary model that values the accuracy and practical utility of de-identified sensitive personal data items. The proper consideration of computational differential privacy relates to the distribution of posterior data [187], which is recovered using the transformed data. Additionally, an adequate level of information privacy [188] is achieved in this context if the probability distribution of the related sensitive personal data does not vary relative to the output of any database or dataset query.

#### 7.1.3. Metrics That Consider Entropy

Relative to the scope of information theory, the entropy designates the degree of uncertainty, which determines the output or outcome of a random variable [189]. The metrics that are determined by entropy generally include the estimated distribution of the reference data, which are computed from the de-identified data. This assertion is true even considering that supplementary descriptive information may be required for a certain metric, such as the original reference data or the parameters used during the data de-identification process. The estimation of sensitive personal data using the available anonymized user data implies that a high degree of uncertainty usually determines a high degree of personal data privacy. Nevertheless, the sensitive personal data may still be re-identified using certain information, which has not been properly de-identified. Thus, the research reported in [190] suggests that the degree of data privacy is quantitavely assessed using cross-entropy, which is also designated as a likelihood. This process considers the estimated and the original data distribution relative to the clustered data, which are derived from the original data.

The authors of [191] described a hybrid model of entropy, which relates to the scope of geographical data privacy. This measures the amount of entropy that can be gathered on a route that is taken, through a series of independent sections. The concept of inherent privacy [192] relates to another metric that is determined by entropy, which considers the number of possible distinct variants relative to a number of binary guesses. Furthermore, mutual information and conditional privacy loss [192,193] represent further entropy-based metrics. The former describes the amount of information that is common to two random variables, which can be calculated as the difference between entropy and conditional entropy. This is also referred to as equivocation, a technique that is particularly useful to calculate the amount of information required to describe a random variable, which implies proper knowledge about another variable that is part of the same dataset. Moreover, the latter metric is determined by a similar structural logic, but it implies the ratio established between the plain data distribution and the amount of information is offered by another variable, which is de-identified in a proper manner.

#### 7.1.4. Metrics Based on the Probability of Success

This class of performance metrics does not refer to the data properties; rather, it considers the success of private information extraction attempts. Low success rates suggest a data privacy model, which ensures a high degree of data protection. Nevertheless, particular users’ personal data may still be illegitimately accessed. The research reported in [194] considered the original data and the estimated data, and a privacy problem was determined by the reconstructed probability of an attribute as the value of the actual computed probability is greater than a specified threshold. This principle is further enriched by the scientific model that was presented in [195] through the description of (d, γ)-privacy, which is determined by additional bounds that are proposed to calculate the ratio between the true and reconstructed probabilities in a more precise manner.

Furthermore, the metric called δ-presence [196] assesses the probability of determining whether the personal data items of an individual are part of certain public datasets. This presumes the existence of a third-party database, which stores the sensitive personal data of all the implied persons. Additionally, it is important to note that hiding failure (HF) [197] represents a data similarity metric, which may be chosen to determine sensitive personal data patterns. This metric is calculated as the ratio between the sensitive patterns, which are detected in the de-identified dataset, and the sensitive data patterns that are detected in the original dataset. Thus, a dataset that is perfectly protected is characterized by a value of zero for this metric.

#### 7.1.5. Metrics Based on the Concept of Error

This class includes metrics that quantify the effectiveness of the sensitive personal data extraction process, generally considering the distance between the original data and the related estimate. Thus, insufficient levels of data privacy are observed relative to small estimate errors. Considering the geographical location privacy, the respective estimation error assesses the correctness of the inference through the computation of the expected distance between the actual location and the estimated location through the utilization of a proper distance metric, such as the Euclidean distance [198]. Moreover, high-dimensional or unstructured data, as an example of data that are collected by mobile background sensors, may be processed through a comparison that considers the traditional performance metrics, which relate to the sensitive private attribute extraction methods using de-identified and original data. Thus, accuracy is a traditionally used metric in this context. It is relevant to note that an important drop in performance constitutes a clear indication that the respective data anonymization technique is valid.

#### 7.1.6. Metrics Based on the Concept of Accuracy

This class includes metrics that assess the accuracy of the inference model. This is based on the principle that inaccurate estimates usually suggest a higher degree of data privacy. The width of the confidence interval designates the quantitative degree of data privacy, considering the estimated interval that includes the valid results [41]. This metric is quantified in percentage form relative to a particular confidence level. Thus, (t, δ) privacy violation [199] offers information concerning the susceptibility of a data classifier’s [200] version public release to constitute a privacy threat, which is determined by the number of training samples that are at the disposition of the adversary algorithm. In essence, the data samples considered to train the model logically connect the publicly available data to the sensitive private data of the reference individuals. Thus, the basic data privacy principles are infringed as it becomes possible to discern sensitive information out of the publicly available data in the case of the persons that are not part of the data training samples.

Considering geographical location data privacy, the size of the region that contains uncertain data determines the lower size of the region, which a certain target user belongs to, and the coverage of the region that contains private data assesses the degree of overlap between a user’s sensitive regions and the region that contains uncertain data [201]. Moreover, another relevant contribution was presented in [202], which presents a possible method for the customization of the data region accuracy, which the end users are part of during the data submission process to an Internet-based service. Consequently, the accuracy of the obfuscated data region is proportional to the degree of data accuracy which is obtained.

#### 7.1.7. Metrics Based on the Concept of Time

This specific type of metrics assess the time that is necessary to extract the required sensitive private data. As an example, relative to geographical location data, the assessment of a reference data privacy technique can be conducted by measuring the longest time interval within which it is possible to successfully break the enforced data privacy, through successful user tracking. This is achieved by calculating the maximum tracking time [203] or the mean time to confusion [204].

## 8. Analytical Discussion Concerning Relevant Research Aspects and Gaps

The comprehensive scientific literature, which was surveyed, suggests that in spite of the progress realized, numerous theoretically fundamental and practical problems require further research be conducted. Consequently, this section analyzes the scientific experience gathered during this scientific research through a discussion concerning the observed relevant scientific research aspects, which require further investigation.

Thus, it is important to observe the correlation between the different sensitive data attributes. This is useful to identify the particular attributes, which may be used to infer the plain text meaning of other sensitive data attributes. As an example, the end user’s geographical location, as determined using the Wi-Fi device, may also suggest valuable data regarding the daily activities that the user attends.

It is important to note that the inferable attributes, which have been extensively analyzed in this paper, may accept diverse sets of values relative to the size and number of attributes, which are specific to any particular subject. As an example, the existence of a medical condition or the gender of a particular individual represent unique individual features, which may be compatible with a binary or limited set of values. Moreover, other personal attributes, such as age, occupational profile, or geographical location, imply different data collection, processing, and anonymization models. Considering the configuration of the attribute output categories and the greater system complexity, which is computationally expensive, it is possible to obtain a superior level of relevant information related to the private data subject. This generally determines a reduced level of personal data privacy. Consequently, considering the problem of personal data protection, it is appropriate to infer that proper data anonymization techniques should be designed, properly implemented, and used. This implies that data anonymization techniques determine a fundamental scope of research, which has not provided a proper solution for all the real-world use cases. Thus, an adequate balance should be determined between the level of generated data anonymization, the implied computational resources, and also the possibility to safely restore the original plain text format of the de-identified data fields, if this is required by legitimate use cases.

The creation of digital data storage mediums and communication channels, together with the continuously increasing computational capacities, has turned the automatic processing of very large databases into a concrete possibility, so that relevant data and correlations are determined orders of magnitudes faster than before. Consequently, the data-driven soft sensors, which are used for data collection in a variety of real-world use cases, should be configured to gather the data that pertain to just the required data field. This ensures that all subsequent processes, which include data anonymization (de-identification), re-identification, and the effective data processing routines, optimally use the available computational resources and generate a pleasant end user experience. This implies the existence of low data request latencies and, ideally, the complete reliability of the related software systems, which is translated through the generation of correct data processing results. It also implies the continued operation of the implied data processing modules. These represent design and implementational details, which are often overlooked in the literature.

Furthermore, the ethical aspects are also important. This relevance is suggested not only by significant regulations, such as the European GDPR [33], or the American HIPAA [76,77], but also by direct research and practical experience. Thus, even if the data are collected through a legitimate process, the generated results may be influenced by inadvertent data alteration factors. These may be either subjective, such as a mistake that is introduced by a human operator, or determined by software bugs or hardware failures. As an example, the authors of this paper designed and implemented an integrated personal health data management system, which uses both hardware [205] and software sensors, to collect data about the physiological parameters that are monitored [206]. Full data privacy is ensured through homomorphic encryption routines, which were illustrated by the authors of [73,74]. The initial incorrect implementation of certain homomorphic encryption routines determined the triggering of false health warnings in the case of individuals who were not suffering from the particular medical conditions. This is just one example, chosen from the authors’ real-world experience, which precisely suggests that the balance between the level of generated data anonymization, the implied computational resources, and the possibility to safely restore the original plain text format of the de-identified data fields, is mandatory during the design and implementation of any such relevant software systems. Such risks are further exacerbated by the software systems, which use deep learning models that do not process the end users’ sensitive personal data in a transparent manner through their internal intermediate layers. Consequently, it may be asserted that fairness in the scope of artificial intelligence (AI) represents a novel, but very intense subject of scientific research, which determines several important real-world aspects, including the implementation of effective personal data privacy models.

### Further Remarks

Built-in sensors in mobile devices collect a variety of real-world data. Consequently, a typical constraint of mobile device-based data computation is robustness. As an example, regarding the interpretation of sensitive personal information, the property designated as “position invariance” may ensure a negligible effect on the algorithmic data processing performance relative to the changes in the position of the end user. More precisely, the algorithmic routines should be able to accurately determine the predefined user attributes’ values, regardless of the actual mobile devices’ position or usage pattern. This represents another practical aspect that is not always properly considered.

It is also relevant to determine whether the end users are involved in the sensitive personal data attributes labeling process. If the answer is affirmative, it is essential to make sure that the respective individual is capable to act in an objective manner, particularly considering certain use cases, such as psychological mood recognition. Furthermore, relative to 3D data that are collected by motion sensors over time, the labeling process may be computationally expensive and complicated. Therefore, the improvement of the existing labeling processes represents an essential research topic, which directly influences the machine-learning-based data processing approaches. Thus, a potential solution may be represented by the consideration of self-supervised learning (SSL) models, a paradigm that favors the training of the feature extraction algorithms through an unsupervised process.

The literature dealing with mobile device sensors suggests that it may be important to assess how internal physical sensors features influence the efficiency and safety of the data collection and anonymization. This is a consequence of the variability in the data collection sensors’ technical specifications and physical features, such as full-scale values, resolution, sampling frequencies, and so on.

Regarding mobile devices, time-based constraints are usually important in the real-time applications and can determine a superior end user experience. Consequently, it is essential that the data anonymization processes not introduce significant overhead relative to the overall computational time efficiency. This represents another conceptual and practical problem, which is not properly addressed in the literature.

It is worth mentioning that an important research aspect concerns the storage of the necessary algorithms. Thus, the collected raw data may be transmitted to powerful cloud-based data processing infrastructures to support the data processing models’ training, which is usually computationally expensive. Therefore, the transmitted data are exposed to more significant risks of unauthorized interception during the transmission phase or to risks of unauthorized access during the data storage phase. Therefore, full data privacy preserving models, such as the homomorphic encryption-based algorithmic routines, may be required in a variety of real-world use cases. It is rather interesting to observe that a preoccupation with full privacy preserving data processing models is not substantial in either the surveyed papers or in the general scientific literature.

The scientific gaps that were identified in the surveyed literature suggest that the preservation of personal data privacy should be ensured, implemented, and quantitatively analyzed through the utilization of a unified data anonymization framework, which includes the required metrics that evaluate the data privacy, as determined by the implemented data anonymization mechanisms. This represents an important but insufficiently approached problem, which may ensure the time-efficient and safe operation of any software system that uses data-driven soft sensors for sensitive private data collection.

## 9. Conclusions and Open Questions

This survey demonstrates that seemingly harmless personal data collection processes may help isolate essential personal data items, which must be protected according to the general technical specifications of relevant data protection regulations, such as GDPR and HIPAA. This paper offers a state-of-the-art survey concerning classic and up-to-date scientific papers, which address the isse of data privacy. The reviewed scientific literature suggests that certain research questions remain unanswered and need further consideration. These include: the detection of correlation between sensitive attributes, efficient data modification algorithms for privacy protection, unified quantitative assessment metrics related to data anonymization methods, and the study of relevant ethical implications.

## Figures and Tables

**Table 1:** Considered scientific literature sources.

Scientific Literature Source	Source Type	Public URL
Science Direct-Elsevier	Digital library	Science Direct (http://www.sciencedirect.com/, accessed on 23 December 2022)
Scopus	Search engine	Scopus (http://www.scopus.com/, accessed on 23 December 2022)
IEEE Xplore	Digital library	IEEE Xplore (http://ieeexplore.ieee.org/Xplore/home.jsp, accessed on 23 December 2022)
ACM Digital library	Digital library	ACM Digital library (http://dl.acm.org/dl.cfm, accessed on 23 December 2022)
Web of science	Search engine	Web of science (https://www.webofknowledge.com/, accessed on 23 December 2022)
Wiley online library	Digital library	Wiley online library (https://onlinelibrary.wiley.com/, accessed on 23 December 2022)
Google Scholar	Search engine	Google Scholar (https://scholar.google.ro/, accessed on 23 December 2022)
Sensors	Digital library	MDPI Sensors Journal https://www.mdpi.com/journal/sensors, accessed on 23 December 2022)
Springer	Digital library	Springer digital library (https://www.springer.com/, accessed on 23 December 2022)
ResearchGate	Scientific social networking	ResearchGate (https://www.researchgate.net/, accessed on 23 December 2022)
Edinburgh library database	Digital library	Edinburgh library database (https://my.napier.ac.uk/Library/, accessed on 23 December 2022)

**Table 2:** Inclusion criteria.

Inclusion Criteria
Papers should be indexed by at least one of the presented scientific paper sources.
Contributions are reported in the period 2010–2022, while relevant older historic papers are also considered.
Papers should fulfill at least one of the search terms, as designated by the title, abstract, and keywords of this survey paper.
Contributions should be published in indexed journals, conference proceedings, or mainstream technical journals.
Surveyed papers should clearly address and answer defined research questions.
A search that considers title, abstract, and full text is sufficient.

**Table 3:** Exclusion criteria.

Exclusion Criteria
Papers that are not written in English.
Duplicated papers, which are found using more than one of the specified scientific literature sources.
Papers with full texts that are impossible to access.
Papers that are only marginally relevant to the usage of data-driven soft sensors, related deep learning models, and data anonymization techniques.

## Data Availability

Not applicable.
